# Dr. Orson S. St. John

**Published:** 1897-08

**Authors:** 


Dr. Orson S. St. John, formerly a resident of Buffalo, died at New York, July 9, 1897, aged 87 years. He was born in Buffalo. His mother's house was the only one left standing at the time Buffalo was burned by the British and the Indians in 1812. He joined the Medical Society of the County of Erie in 1831.

His last public appearance in Buffalo was many years ago, when he read a paper before the Buffalo Historical Society. His only relatives in this city are the members of the Sidway family. He was a younger brother of the late Mrs. Sidway, the mother of Franklin Sidway, of this city, and was well known among old residents of Buffalo.

After preliminary study in Buffalo, Dr. St. John was educated in law in Cleveland and Cincinnati, O., and was graduated in medicine in Philadelphia. Possessed of independent means, he practised little in either profession, but for half a century had devoted himself to literature and scientific investigation, the latter especially directed to the origin of life and celestial mechanics and resulting in the discovery of many now well-known principles. Dr. St. John was a deep student and extensive traveler and was widely known in collegiate and scientific circles in Europe and America. His wife died several years ago and his home since then, when in this country, had been with his daughter, Mrs. Andrews. G. C. St. John, of New York, is his son. His body was taken to Ohio for burial.

